# Supplementary material for: Real-Time Longitudinal Evaluation of Tumor Blood Vessels Using a Compact Preclinical Fluorescence Imaging System
Source: Biosensors (Basel). 2021 Nov 23;11(12):471. doi: 10.3390/bios11120471 (PMC8699707; doi:10.3390/bios11120471)
Supplement: Supplementary file 1 [file biosensors-11-00471-s001.zip › biosensors-1417297_supplemental document.pdf]

Supplymentary Information

Real-time longitudinal evaluation of tumor blood vessels using a compact preclinical fluorescence imaging system

Hoibin Jeong[1,5], Song-Rae Kim[1,5], Yujung Kang[2], Huisu Kim[2], Seo-Young Kim[1,3], Su-Hyeon Cho[1], Kil-Nam Kim[1,4,*]

[1]*Chuncheon Center, Korea Basic Science Institute (KBSI), Chuncheon 24341, Republic of Korea*
[2]*Vieworks, Anyang 14055, Republic of Korea*
[3]*Division of Practical Application, Honam National Institute of Biological Resources, Mokpo 58762, Republic of Korea*
[4]*Department of Bio-Analytical Science, University of Science and Technology, Daejeon 34113, Republic of Korea*
[5]*These authors contributed equally to this work*
[*] *knkim@kbsi.re.kr (K.-N. Kim).*

Supplementary figures

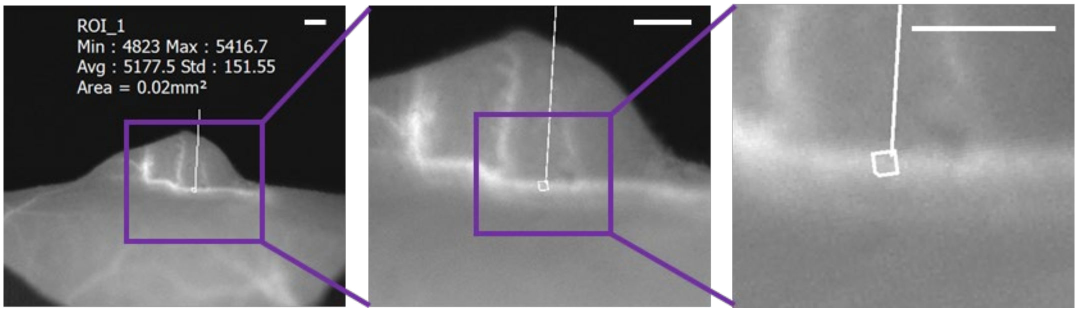

Figure S1. ROI drawn to include only the blood vessel area. Purple rectangles are enlarged on the right-hand side. Scale bar, 1 mm

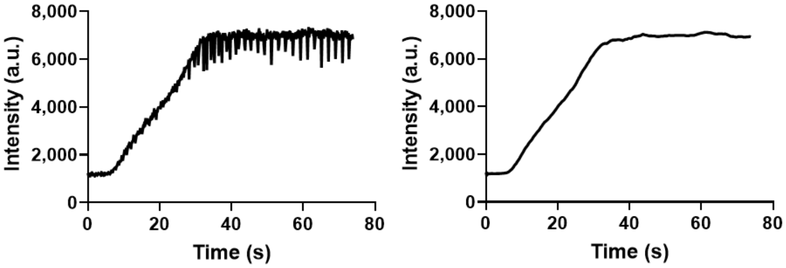

Figure S2. ICG dynamics of a pixel on tumor blood vessel when mouse movement due to breathing is reflected (left) and when artifacts are removed using Savitzky-Golay Filter (right).

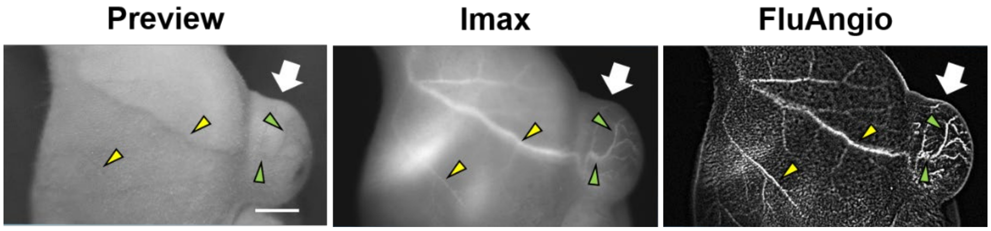

Figure S3. Preview (photograph), Imax, and FluAngio maps in an A431 tumor-bearing mouse. White arrows indicate the A431 tumor. Yellow or green arrowheads indicate blood vessels in the tumor periphery or within the tumor, respectively. Scale bar, 5 mm.

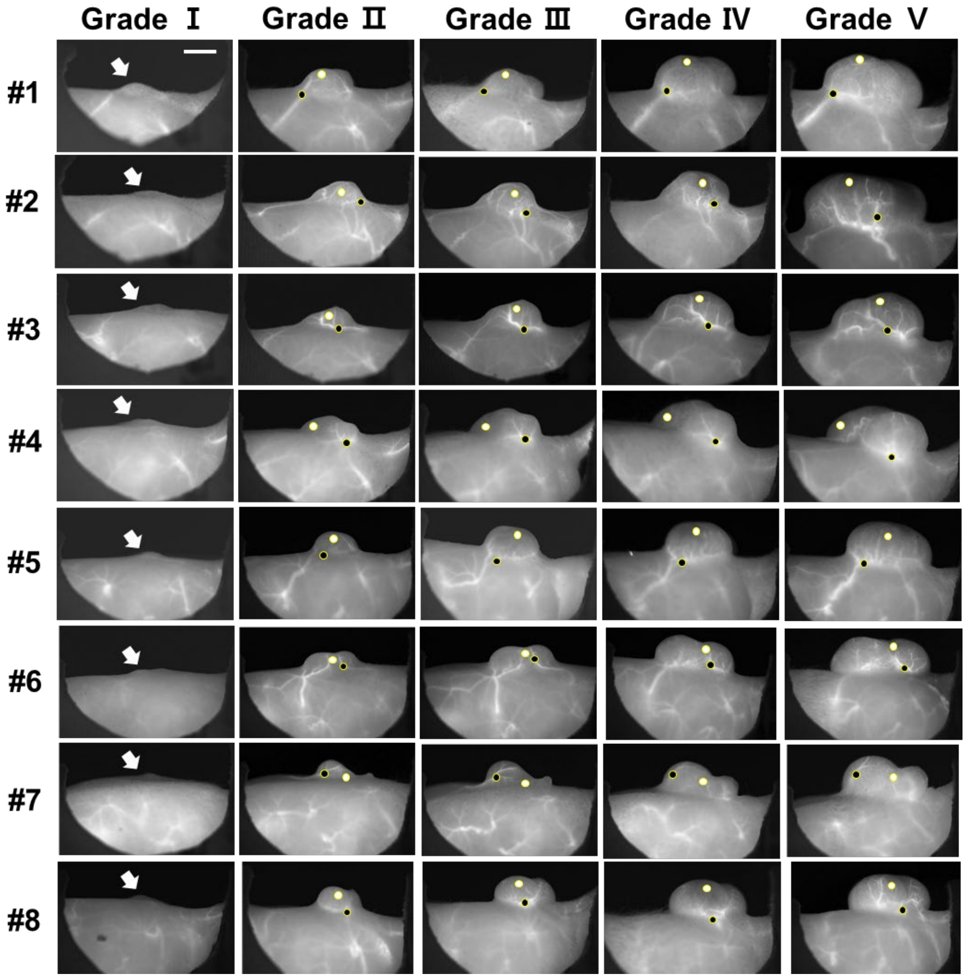

Figure S4. Fluorescence images of A431 tumors for each grade determined based on the tumor size. The white arrow on Grade I indicates the location of the small palpable tumor. In Grades II, III, IV, and V, black circles indicate blood vessels and white circles indicate outer areas. Tumor #3 is the one in Figure 4A. Scale bar, 5 mm.

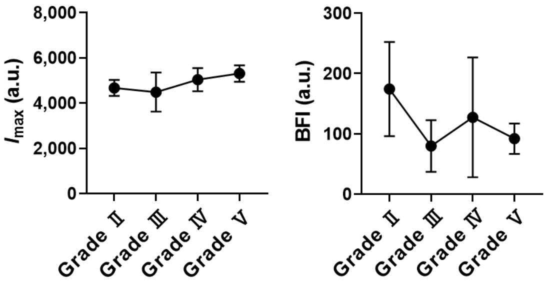

Figure S5. Changes in I_{max} and BFI when the entire tumor area is designated as an ROI by grade. Data are the mean ± SD for n = 8 mice per grade.

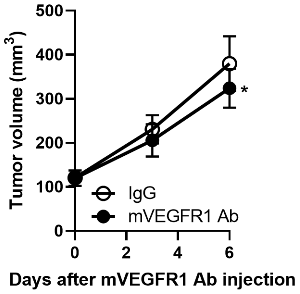

Figure S6. Growth of A431 tumors in mice injected with IgG or mVEGFR1 Ab twice every three days. Data are the mean ± SD for $n \geq 7$ mice per group. *, $P < 0.05$ determined via two-way ANOVA.

Supplementary tables

	**Ultrasound Doppler Flowmetry**	**Laser Doppler Flowmetry**	**Laser Speckle Contrast Imager**	**VISQUE® InVivo Smart-LF**
Temporal Resolution	A few seconds	A few minutes	a few seconds	Once at a time
Spatial Resolution	-	1 mm – 0.1 mm	tens of micrometer	27 µm (@3✕)
Imaging Depth	A few mm depth	Less than 1 mm depth	Less than 1 mm depth	According to the FL dye
Imaging Target	RBC	RBC	RBC	Fluorescence dye in blood

Table S1. Comparison of VISQUE® InVivo Smart-LF equipment and other optical *in vivo* imaging systems for small animals with resolution, imaging depth and target.

Model	VSP-4MU
Resolution	2048(H) ✕ 2048(V)
Sensor Type	Backside Illuminated Scientific CMOS Image Sensor

Pixel Size	6.5 µm ✕ 6.5 µm
Optical Format	1.2”
Cooling Method	Thermoelectric Peltier Cooling
Cooling Performance	50℃ below ambient temperature
Quantum Efficiency	Max. 94 % @ 550 nm
Full Well Capacity	54 ke^-
Max. Frame Rate	37 fps
Exposure Time (1µs step)	11 µs ~ 6 s
Binning	2 ✕ 2, 4 ✕ 4
Output Format	Mono 16
Lens Mount	C-mount
Interface	USB 3.0

Table S2. Specifications for the sCMOS camera, VSP-4MU.

**Model**	**VISQUE® InVivo Smart LF**
Weight and Dimension	About 20 kg (44 lbs), 40 cm ✕ 40 cm ✕ 57 cm
Sensor	1.2“ Backside Illuminated sCMOS
Cooling	-20℃, Thermoelectric Peltier Cooling
Resolution (H ✕ V)	1824 ✕ 1824
Pixel Size	6.5 µm ✕ 6.5 µm
Exposure Time	25 ms – 30 mins
Maximum Frame Rate	37 fps
Digital Output	16 bit
Binning	1 ✕ 1, 2 ✕ 2, 4 ✕ 4
Fluorescence Light Source	LED
Fluorescence Emission Filter	Up to 9, Optional
Lens Control	Motorized Iris / Zoom / Focus
Zoom (Field of View, H ✕ V)	15 cm x 15 cm (1✕) – 5 cm x 5 cm (3✕)
Stage Type	Sliding stage, Up to 3 mice
Accessory	Heating stage, Anesthesia Ventilator Adaptor

Table S3. Specifications for the VISQUE® InVivo Smart-LF equipment.
